# Protocol for the Systematic Quantitative Ultrastructural Analysis of Mitochondria in Cardiac Tissue

**DOI:** 10.3390/mps8040087

**Published:** 2025-08-02

**Authors:** Rebecca Schönmehl, Lina Winter, Daniel H. Mendelsohn, Wing-Hoi Cheung, Ronald Man Yeung Wong, Steffen Pabel, Samuel Sossalla, Christoph Brochhausen

**Affiliations:** 1 Institute of Pathology, Medical Faculty Mannheim, Heidelberg University, 68167 Mannheim, Germany; rebecca.schoenmehl@medma.uni-heidelberg.de (R.S.);; 2 Department of Neurology, LMU University Hospital, LMU Munich, 81377 Munich, Germany; 3 Department of Orthopaedics and Traumatology, The Chinese University of Hong Kong, Hong Kong, China; 4 Department of Internal Medicine II, University Medical Center Regensburg, 93053 Regensburg, Germany; 5 German Center for Cardiovascular Research (DZHK), Partner Site Rhein Main, 61231 Bad Nauheim, Germany

**Keywords:** mitochondria, electron microscopy, ultrastructure, cardiomyocytes, quantitative image analysis

## Abstract

Mitochondria play a crucial role in adapting to fluctuating energy demands, particularly in various heart diseases. In addition to functional analyses such as the measurement of ROS or ATP, analysis of mitochondrial ultrastructure can be used to draw further conclusions about their functions and effects in tissue. In this protocol, we introduce a set of measurements to compare the ultrastructural and functional characteristics of human left ventricular mitochondria, using transmission electron microscopy (TEM). Measured parameters included mean size in µm^2^, elongation, count, percental mitochondrial area in the measuring frame, and a conglomeration score. We also introduce a novel method of defining hydropic mitochondria as a comparable evaluation standard. With this cluster of measurement parameters, we aim to contribute a protocol for studying human mitochondrial morphology, distribution, and functionality.

## 1. Introduction

Mitochondria are eucaryotic cell organelles that play an essential role in various cellular processes, such as energy production, Ca^2+^ homeostasis, lipid metabolism, and apoptosis. The ultrastructure of mitochondria is characterized by a dual-phospholipid membrane structure. The outer membrane serves as an external barrier and plays a key role in mitochondrial fission and fusion processes that also involve the inner membrane. The inner membrane, whose protrusions are known as cristae, contains protein complexes that facilitate efficient ATP synthesis via oxidative phosphorylation. The matrix contains a variety of enzymes critical for the citric acid cycle, as well as mitochondrial DNA and ribosomes, which are crucial for mitochondrial biogenesis and function [1].

Mitochondrial dysfunction can result in several diseases, notably mitochondriopathies, a rare but severe group of conditions that compromises cellular energy metabolism and manifests in various organs [2]. Furthermore, mitochondrial dysfunction is linked to other conditions, including cancer as well as neurodegenerative and cardiovascular diseases [3]. Interestingly, the ultrastructure of mitochondria can provide information about their functionality and may be altered in pathologic states [4]. For instance, megamitochondria have two- to threefold the normal size and have been reported to occur in liver diseases [5]. Although the exact mechanisms need to be elucidated, it is assumed that swelling occurs as a compensatory response during cellular stress [4]. Another common reason for mitochondrial enlargement is mitochondrial swelling due to the entry of water [6]. On the other hand, fragmentation of the mitochondrial network has been observed in cancer [7].

Furthermore, neurodegenerative diseases are linked to mitochondrial dysfunction. In this context, Keskinoz et al. have demonstrated that in Alzheimer’s disease, there is a significant number of teardrop-shaped mitochondria. This ultrastructural feature might indicate alterations in mitochondrial fission processes [8].

Despite the growing evidence that mitochondrial dysfunction is crucial in the pathogenesis of many diseases, there exists no standardized method for the quantitative evaluation of mitochondrial ultrastructure. However, it remains a challenge to close this methodological gap, since the ultrastructure of mitochondria presents differently in various tissues, for instance, in terms of cristae morphology [9]. Cardiac tissue contains elongated mitochondria with closely stacked, roughly parallel cristae. Similarly, kidney mitochondria show lamellar and locally parallel cristae. However, liver mitochondria differed significantly compared to heart and kidney. Specifically, they presented less homogenously, being irregular and not organized in parallel stacks [10]. Therefore, a tissue-specific approach seems appropriate in the quantitative analysis of mitochondrial phenotypes.

Previous research has developed several approaches to assess mitochondrial ultrastructures and thus their functionality. In this context, the measurement of cristae angles can provide insights into the structural integrity of mitochondria, particularly under stress or disease conditions [11,12]. Additionally, the roundness of mitochondria is used to quantify how the hydropic shape relates to mitochondrial efficiency and overall cell health, with a more rounded appearance often represent potential distress or dysfunction [13,14]. Furthermore, surface density provides a measure of the mitochondrial membrane area available for bioenergetic processes per unit volume of tissue, crucial for assessing metabolic capacity. Volume density offers insights into the proportion of cell or tissue volume occupied by mitochondria, indicating the overall mitochondrial abundance and potential energy output of the cells [15]. In previous retrospective studies, we already determined some parameters for the ultrastructural evaluation of mitochondria, such as the conglomeration score. This score evaluates the spatial distribution and degree of mitochondrial clustering. This is of interest, as in stressed or diseased cells (e.g., ischemia, heart failure), mitochondria may fragment, aggregate, or collapse into clusters, disrupting normal cellular function [16].

In the present report, we aim to contribute a protocol for studying mitochondrial morphology, distribution, and functionality. Specifically, we introduce a new method for defining hydropic mitochondria as a comparable evaluation standard.

## 2. Experimental Design

### 2.1. Materials

1. Cardioplegic solution (Dr. Franz Köhler Chemie GmbH, Bensheim, Germany, CUSTODIOL^®^);
2. Liquid nitrogen;
3. Karnovsky Fixative (aqueous buffered glutaraldehyde solution);
4. Sodium Cacodylate 0.1 M (ScienceServices, Munich, Germany Article#E12310);
5. Osmiumtetraoxide, 2% (ScienceServices, Munich, Germany Article#19100);
6. Double-distilled water;
7. Ethanol (Honeywell Chemicals, Offenbach, Germany, Article#32205-2.5L-GL);
8. Propylene oxide (Sigma Aldrich, Darmstadt, Germany, Article#110205-1L);
9. EPON resin (ScienceServices, Munich, Germany Article#14120);
10. Aqueous 2% uranyl acetate solution (Serva, Heidelberg, Germany, Article# 77870.02);
11. Two-percent lead citrate solution (Leica, Wetzlar, Germany, Article# 16707235).

### 2.2. Equipment

- LYNX microscopy tissue processor (Reichert-Jung, Wetzlar, Germany);
- Leica Ultracut S Microtome (Leica-Reichert, Wetzlar, Germany);
- LEO 912AB electron microscope (Zeiss, Oberkochen, Germany);
- Side-mounted camera with a 2k × 2k resolution and iTEM software version 5.2 (OSIS, Muenster, Germany);
- Graphics tablet Huion Inspiroy Dial 2 (Huion, Shenzhen, China);
- RADIUS version 2.2 (EMSIS, Muenster, Germany);
- Excel version 1808 (Microsoft, Redmond, WA, USA);
- GraphPad Prism version 9.5.1 (GraphPad Software, San Diego, CA, USA).

## 3. Procedure

### 3.1. Sample Acquisition and Evaluation

#### 3.1.1. Sample Acquisition and Handling

- Acquire representative tissue samples from the left ventricle of the human heart in open heart surgery, while being mindful of the presence of stents and fibrosis.
- After removal, transfer the samples into a cardioplegic solution (CUSTODIOL^®^, Dr. Franz Köhler Chemie GmbH, Bensheim, Germany) at 4 °C.
- Transport the samples out of the operating room into the laboratory to snap-freeze them in liquid nitrogen.

#### 3.1.2. Sample Preparation for TEM

- Thaw samples in Karnovsky Fixative (an aqueous buffered glutaraldehyde solution) for at least 48 h.
- Embed the samples (post fixation with osmium tetroxide, dehydration, infiltration with EPON) in the LYNX microscopy tissue processor (Reichert-Jung, Wetzlar, Germany) following the embedding protocol (Section 5.1).
- Cut semi-thin sections (0.75 µm) using the Leica Ultracut S Microtome (Leica-Reichert, Wetzlar, Germany).
- Stain the semi-thin sections with toluidine blue and basic fuchsin following the staining protocol (Section 5.2).
- Select relevant areas.
- Trim away non-relevant areas of the sample block as needed.
- Cut ultra-thin sections (80 nm) using the Leica Ultracut S Microtome (Leica-Reichert, Wetzlar, Germany).
- Contrast the ultra-thin sections with aqueous 2% uranyl acetate and 2% lead citrate solutions for 10 min each using the contrasting protocol (Section 5.3).

#### 3.1.3. Image Acquisition and Analysis

**Figure 1:**
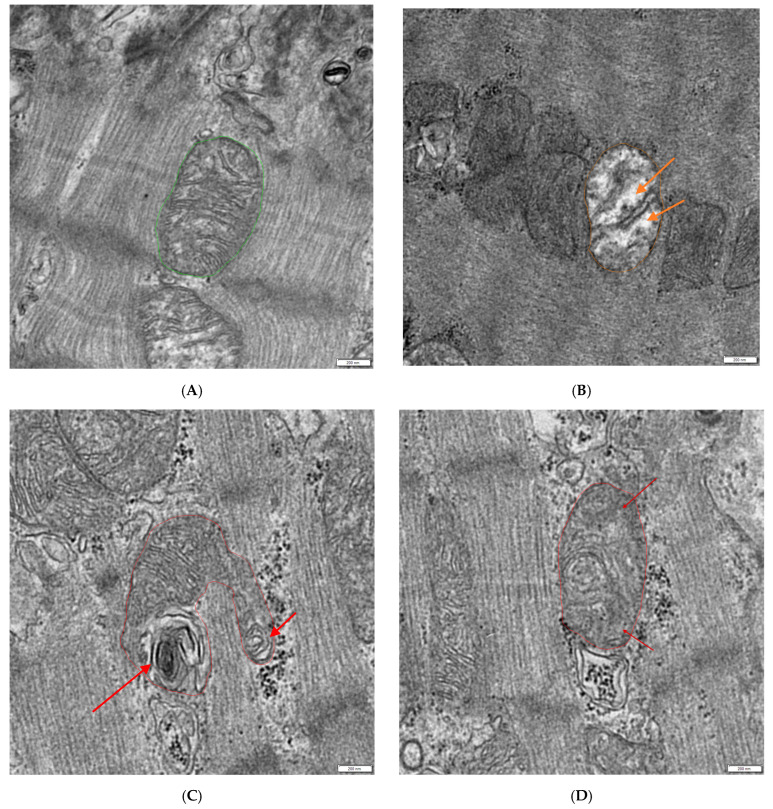
Comparison of mitochondria ultrastructure. (**A**) Normal mitochondria in green; (**B**) hydropic mitochondria in orange with reduced matrix density (orange arrows). (**C**,**D**) Otherwise defective mitochondria in red (lamellar structures arising from mitochondria (red arrows); loss of cristae without the loss of matrix density (dark red arrows)); the scale bar in the right lower corner equals 200 nm.

- For sample documentation, use the 2k × 2k side-mounted camera of a LEO 912AB electron microscope (Zeiss, Oberkochen, Germany) with iTEM software version 5.2 (OSIS, Muenster, Germany) to depict the mitochondria of the cardiac tissue at 10,000× magnification.
- For the evaluation of ultrastructural images, open the acquired images using the RADIUS version 2.2 software (EMSIS, Muenster, Germany).
- Select areas for evaluation (measuring frame), excluding interstitial space and artifacts.
- Use the Huion Inspiroy Dial 2 graphic tablet to individually annotate the mitochondria using the freehand polygon function.
- Obtain the measured values of area, perimeter, and sphericity from RADIUS version 2.2 (EMSIS, Muenster, Germany).
- Manually evaluate which mitochondria appear normal, hydropic (decreased matrix density with loss of cristae), or otherwise defective (lamellar structures arising from mitochondria or the loss of cristae without the loss of matrix density) and count them (see Figure 1).

OPTIONAL STEP

For borderline cases where it is unclear whether the mitochondria are normal or hydropic, use the following steps. It is critical to calculate the base line for each affected image, due to possible different exposure times of the depicted region.

**Figure 2:**
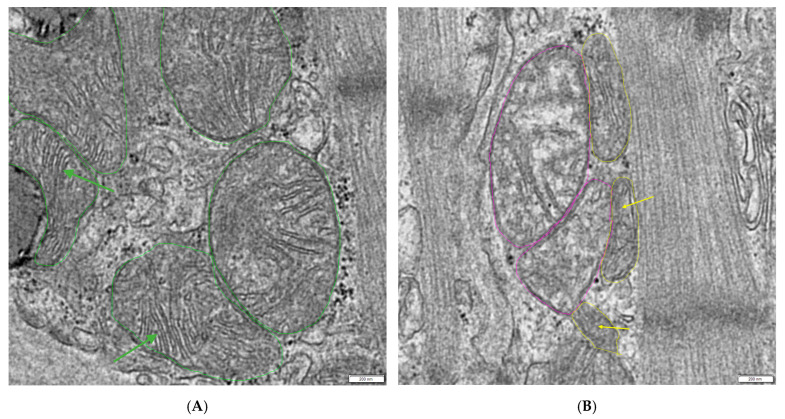
Mean gray value evaluation of mitochondria. (**A**) Selection of healthy mitochondria as decision support to classify (**B**) borderline mitochondria as hydropic or normal: green-labeled normal mitochondria with dense matrices and clearly visible cristae (green arrows), suitable for calculating a base line, yellow-labeled normal mitochondria, unsuitable for calculating a base line due to their lack of cristae definition (yellow arrows), and pink-labeled borderline mitochondria. The scale bar in the right lower corner equals 200 nm.

- Obtain the mean gray value of the annotated mitochondria using the RADIUS version 2.2 software (EMSIS, Muenster, Germany).
- Select 3 normal mitochondria with dense matrices and clearly visible cristae (see Figure 2A).
- Use Excel version 1808 (Microsoft, Redmond, WA, USA) to calculate the mean gray value of these 3 mitochondria (base line) and calculate the cut off limit using the multiplication factor 1.1 (1.1 × base line = cut off limit).
- If the mean gray value of the borderline mitochondria is equal or greater than the calculated cut off limit, count them as hydropic.

#### 3.1.4. Calculation of the Conglomeration Score and Statistical Analysis

- Use Excel version 1808 (Microsoft, Redmond, WA, USA) to calculate the conglomeration score (CS) (Section 3.3). If a measuring frame (MF) shows a cluster of mitochondria that is no longer situated in between but is overgrowing the muscle fibers, this MF is counted as conglomerated. Depending on the size of the MF, the area that shows the conglomeration is weighted proportionally to the total analyzed area per patient.
- Use Excel version 1808 (Microsoft, Redmond, WA, USA) to count the total number of annotated mitochondria and the number of normal, hydropic, and otherwise defect mitochondria;
- Transfer your raw data to GraphPad Prism version 9.5.1 (GraphPad Software, San Diego, CA, USA) using a separate column data table for each measured parameter.
- Conduct either a *t*-test (2 groups) or a one-way ANOVA (more than 2 groups) for each data table.
- Create a new column table for the correlation of measurement parameters. Enter the data so that the individual measurement parameters form columns and ensure that the patient data in each column is always entered in the same order of rows (example: from top to bottom, patient 1 to patient 10).
- Create a correlation matrix for this table.

### 3.2. Conglomeration Score Calculation

Conglomeration score = 100: Σ A (all evaluated MF) × Σ A (MF with conglomeration)

Example: MF1 = 120 µm^2^, conglomeration = yes; MF2 = 300 µm^2^, conglomeration =
no; and MF3 = 80 µm^2^, conglomeration = yes.                   

CS = 10: A(MF1 + MF2 + MF3) × A(MF1 + MF3) = 100: 500 µm^2^ × 200 µm^2^ = 40

### 3.3. Definition of the Multiplication Factor

The multiplication factor was defined as 1.1 after comparing the mean gray value of hydropic mitochondria with healthy mitochondria. After analyzing more than 1500 mitochondria per group, we found that all hydropic mitochondria were at least 10% lighter in their mean gray value than healthy mitochondria.

## 4. Expected Results

This protocol was used in previous retrospective studies. The mitochondria of patients with sinus rhythm, arterial fibrillation, and dilatative and ischemic cardiomyopathy were analyzed using this protocol for the systematic quantitative ultrastructural analysis of cardiac tissue. Overall, 22,781 mitochondria were evaluated using the protocol (5870 belonged to patients with SR, 8173 to patients with AF, 4507 to patients with DCM, and 4231 to patients with ICM). These 22,781 mitochondria contained 18,086 healthy, 2395 hydropic, and 2300 otherwise defective mitochondria. For around 1300 mitochondria that were initially classified as borderline, the optional step in Section 3.1.3. was used to determine whether they were healthy or hydropic.

This quantitative ultrastructural analysis protocol allows for the systematic assessment of mitochondrial morphology, size, distribution, and functional state in human cardiac tissue. Using this approach, researchers can expect to obtain detailed measurements of mitochondrial area, perimeter, sphericity, and conglomeration scores across multiple measuring frames, alongside counts of normal, hydropic, and otherwise defective mitochondria.

In healthy cardiac tissue, mitochondria typically appear more elongated, with an electron-dense matrix and clearly visible, regularly arranged cristae [16,17]. The conglomeration score should remain low, reflecting an even distribution of mitochondria between muscle fibers [17]. In contrast, pathological samples (e.g., from ischemic or failing hearts) may show increased numbers of hydropic mitochondria (swollen, with loss of cristae and decreased matrix density), elevated conglomeration scores—due to mitochondrial clustering or overgrowth over muscle fibers—and a shift in shape toward rounder, fragmented forms [18,19].

While the protocol provides robust tools for comparing the mitochondrial ultrastructure across conditions, it is important to emphasize that no single cutoff value or measurement can serve as an absolute indicator of mitochondrial health or pathology. For example, defining “healthy” mitochondria solely based on matrix electron density and cristae visibility risks overlooking subtle or borderline pathological changes. Therefore, users are cautioned that the calculated cutoff values (e.g., for distinguishing hydropic mitochondria based on gray value thresholds) should not be considered a universal or definitive standard. Rather, they are context-dependent and require careful validation within each experimental setup.

Researchers applying this protocol should remain aware of the potential for misclassification if selection criteria are too rigid or if the evaluated “healthy” mitochondria do not adequately represent the normal state of mitochondria in the sample. To ensure reliable and meaningful results, we recommend using multiple parameters in combination and interpreting findings within the biological and pathological context of the tissue under investigation. Furthermore, since the method for ultrastructural analysis shown in this protocol was developed for two-dimensional images, it only represents the state of mitochondria at one level of heart tissue. In order to further understand mitochondrial changes with regard to their overall shape or cristae, as well as distribution and functionality, we recommend considering additional three-dimensional observations [5,11].

## 5. Reagents Setup

### 5.1. Embedding Protocol

The previously fixated samples were transferred into the sample holder of the LYNX microscopy tissue processor (Reichert-Jung, Wetzlar, Germany) and then underwent the following steps for tissue embedding (see Table 1):

### 5.2. Staining Protocol

- Place the glass slide containing the semi-thin sections on a heat plate at 80 °C.
- Apply 0.1% aqueous toluidine blue solution on the slide and stain for 60 s.
- Rinse off the toluidine blue with double-distilled water.
- Place the glass slide back onto the heat plate.
- Apply 0.1% basic fuchsin solution on the slide and stain for 30 s.
- Rinse of the basic fuchsin with double-distilled water.
- Dry the slide on the heat plate.

### 5.3. Contrasting Protocol

- Line the bottom of two Petri dishes with parafilm.
- Cover a Petri dish lid with aluminum foil and place it on one of the Petri dish bottoms (Petri dish 1).
- Place one of the Petri dish bottoms into a slightly larger Petri dish, place sodium hydroxide pellets in the space between, and cover with a lid (Petri dish 2).
- Prepare three 100 mL beakers containing double-distilled water.
- Put a drop of uranyl acetate onto the parafilm-covered surface of Petri dish one and a drop of lead citrate in Petri dish 2.
- Place the copper grid containing the ultra-thin section on the uranyl acetate drop with the sample side down.
- Cover the Petri dish with the lid and wait for 10 min.
- Wash the copper grid by carefully dunking the grid vertically into the water multiple times for each beaker.
- Dry the residual water by blotting with a piece of filter paper at the edge of the grid.
- Place the copper grid containing the ultra-thin section on the lead citrate drop with the sample side down.
- Cover the Petri dish with the lid and wait for 10 min.
- Wash the copper grid by carefully dunking the grid vertically into the water multiple times for each beaker.
- Dry the residual water by blotting with a piece of filter paper at the edge of the grid.
- Place grids into a new Petri dish to dry and cover it with a lid.

## Figures and Tables

**Table 1:** Embedding protocol.

Vial	Chemical	Time
1	Sodium Cacodylate 0.1 M	30 min
2	Sodium Cacodylate 0.1 M	30 min
3	Sodium Cacodylate 0.1 M + 2% OsO_4_ (1:1) (final concentration 1%)	120 min
4	Sodium Cacodylate 0.1 M	30 min
5	Double-distilled water	10 min
6	Double-distilled water	10 min
7	Double-distilled water	10 min
8	Ethanol 50%	15 min
9	Ethanol 70%	15 min
10	Ethanol 90%	15 min
11	Ethanol 95%	15 min
12	Ethanol 100%	15 min
13	Ethanol 100%	15 min
14	Ethanol 100%	15 min
15	1,2-Propylene oxide	30 min
16	1,2-Propylene oxide	30 min
17	1,2-Propylene oxide + EPON (1:1)	180 min
18	EPON	600–900 min

## Data Availability

Data are contained within the article.
